# Up-regulated SLC25A39 promotes cell growth and metastasis via regulating ROS production in colorectal cancer

**DOI:** 10.7150/jca.98844

**Published:** 2024-09-16

**Authors:** Wentao Zhang, Zhigao Ou, Ting Tang, Tian Yang, Yubo Li, Hao Wu, Li Li, Ming Liu, Li Niu, Jianjun Zhu

**Affiliations:** 1 Department of Medical Cellular Biology and Genetics, School of Basic Medical Science, Shanxi Medical University, Taiyuan, Shanxi, China.; 2 Department of Pathophysiology, School of Basic Medical Science, Shanxi Medical University, Taiyuan, Shanxi, China.; 3 Department of Medical Cellular Biology and Genetics, School of Basic Medical Science, Shanxi Medical University, First Hospital of Shanxi Medical University, Taiyuan, Shanxi, China.

**Keywords:** SLC25A39, colorectal cancer, tumor growth, metastasis, immunotherapy

## Abstract

**Background:** The mitochondrial transporter SLC25A39 has been implicated in the import of mitochondrial glutathione (mGSH) from the cytoplasm, crucial for mitigating oxidative stress and preserving mitochondrial function. Despite the well-established involvement of mitochondria in cancer, the functional impact of SLC25A39 on CRC progression remains elusive.

**Methods:** The mRNA and protein expressions were detected by PCR, immunohistochemistry, and Western blot, respectively. Cell activity, cell proliferation, colony formation, and apoptosis were measured by CCK8 assay, EdU incorporation assay, plated colony formation assay, and flow cytometry, respectively. Cell migration was detected by wound healing and transwell chamber assay. The tumor microenvironment (TME), immune checkpoint molecules, and drug sensitivity of CRC patients were investigated using R language, GraphPad Prism 8 and online databases.

**Results:** Here, we report a significant upregulation of SLC25A39 expression in CRC. Functional assays revealed that overexpression of SLC25A39 promoted CRC cell proliferation and migration while inhibiting apoptosis. Conversely, SLC25A39 knockdown suppressed cell growth and migration while enhancing apoptosis in vitro. Additionally, reduced SLC25A39 expression attenuated tumor growth in xenograft models. Mechanistically, elevated SLC25A39 levels correlated with reduced reactive oxygen species (ROS) accumulation in CRC. Furthermore, bioinformatic analyses unveiled the high SLC25A39 levels was associated with decreased expression of immune checkpoints and reduced responsiveness to immunotherapy. Single-cell transcriptomic profiling identified diverse cellular expression patterns of SLC25A39 and related immune regulators. Lastly, drug sensitivity analysis indicated potential therapeutic avenues targeting SLC25A39 in CRC.

**Conclusion** Our findings underscore the pivotal role of SLC25A39 in CRC progression and suggest its candidacy as a therapeutic target in CRC management.

## Introduction

Colorectal cancer (CRC) ranks as the third most prevalent and the second most lethal malignancies among all cancers 1. There has been a two-fold increase in CRC cases and deaths worldwide over the past three decades 2. By 2022, China was predicted to have 592,232 new cases of CRC and 309,114 new deaths 3. Therefore, understanding the mechanistic underpinnings of CRC progression is imperative. Mitochondria, pivotal in regulating oxidative stress, metabolism, signaling, and cell fate, emerge as crucial mediators in tumorigenesis, growth, metastasis, and survival 4. Mutational perturbations in mitochondrial enzymes precipitate the accumulation of oncometabolites, fostering cancer initiation. Moreover, mitochondrial metabolic reprogramming could facilitate tumor growth and survival 5, 6. Concurrently, redox homeostasis, mitochondrial biogenesis, and mitophagy intricately contribute to tumor dynamics, underscoring the multifaceted roles of mitochondria in CRC pathogenesis and progression.

The reduced glutathione (GSH), as a key antioxidant, is synthesized exclusively in the cytosol through two ATP-dependent enzymes, namely γ-glutamyl-cysteine ligase (GCL), which is comprised of two independent subunits GCLC and GCLM; and glutathione synthetase (GSS) 7, 8. However, mitochondria are devoid of GSH synthesis, whose uptake depends on special transporters. Notably, mitochondrial GSH (mGSH) constitutes a predominantly reduced pool, comprising 10-15% of the total GSH, with concentrations akin to the cytosol (10 to 14 mM) 8. Recently, Wang et al. have unveiled SLC25A39 as an important mitochondrial membrane carrier orchestrating mGSH import 9-11, pivotal for maintaining cellular function and mitigating mitochondrial dysfunction 8, 12. However, the precise impact of aberrant SLC25A39 expression on cellular function in cancer remains unexplored, despite its pivotal role as the primary mitochondrial GSH transporter. Understanding the implications of SLC25A39 dysregulation in tumor biology is paramount for elucidating novel therapeutic avenues in cancer management.

In our study, we observed an oncogenic role of SLC25A39 in CRC cell proliferation and migration, and metastasis, potentially mediated by reactive oxygen species (ROS) accumulation. Further bioinformatics analysis suggested that patients with low SLC25A39 expression might exhibit heightened sensitivity to immunotherapy. These findings underscore the clinical relevance of SLC25A39 as a potential therapeutic target in CRC management.

## Materials and methods

### Cell Culture and tissue collection

CRC tumor tissues and adjacent peritumor tissues were collected from 49 patients who had undergone surgery at the Affiliated Hospital of Shanxi Medical University, and informed consent were obtained from the patients who participated in the present study. Another 8 resected CRC and adjacent peritumor tissues were collected and stored in liquid nitrogen for subsequent mRNA and protein level analysis. The eligibility criteria for CRC patient recruitment were set as follows: (1) histologically-confirmed colorectal carcioma (CRC); (2) receiving surgical resection; (3) availability of complete clinical information; (4) no preoperative anticancer treatment; (5) no history of other malignancy; and (6) alive at least 1 month after surgery. This study was performed with approval from the Ethics Committee of Shanxi Medical University (Permission number: 2020918). In addition, RKO and DLD-1 cell lines were purchased from the Shanghai Cell Bank of the Chinese Academy of Sciences (Shanghai, China). RKO and DLD-1 cells were routinely cultured at 37˚C and 5% CO_2_ in DMEM medium supplemented with 10% fetal bovine serum (FBS).

### Public dataset collection

Bulk RNA-Seq data for 449 COAD samples were retrieved from the TCGA-COAD cohort (http://www.cancer.gov/tcga), while clinical and pathological information was obtained from UCSC Xena (http://xena.ucsc.edu). In this study, samples lacking complete clinical pathological data or with survival times of less than 1 month were excluded. Finally, 408 CRC samples and 41 normal samples were enrolled and analyzed in the present study. Additionally, GSE41258, comprising 186 tumor samples and 54 healthy samples, was utilized for further analysis.

### Immunohistochemistry (IHC)

IHC analysis and quantification of IHC staining were conducted following established protocols^13^. The protein expression of SLC25A39 was evaluated by two independent pathologists who were blinded to the clinical data of the patients. A proportion score, which represents the proportion of positive tumor cells, was assigned as follows: < 10%, 0; 10 to 25%, 1; 26 to 50%, 2; 51 to 75%, 3; and > 75%, 4. An intensity score, which represents the intensity of the positive tumor cells, was assigned as follows: 0 (no staining), 1 (intensity lower than positive control), 2 (intensity equal to positive control), 3 (intensity higher than positive control). The total score = proportion score × intensity score. Primary antibodies used in this study are listed in Supplementary Table S1. A 100 μL aliquot of secondary antibody from the Anti-mouse/rabbit Maxvision-HRP Kit (Cat. No. 5010) was applied. Subsequently, slides were stained with DAB solution and counter stained with hematoxylin.

### Quantitative Real-Time PCR (qRT-PCR)

Total RNA was isolated from cultured CRC cells or CRC tumor and peritumor tissues and reversely transcribed. qRT-PCR was performed as previously reported^14^. The 2^-ΔΔCt^ algorithm was applied to quantify the relative mRNA expression. β-actin was used as an internal control. Primer sequences used for qPCR assay were listed in Supplementary Table S2.

### Western blot

Western blotting was performed based on regular procedures. For cultured cells, the cells were lysed in ice-cold RIPA buffer (Beyotime, China). For tissues, the tissues were ground with liquid nitrogen, and the collecting powdered tissue was lysed in ice-cold RIPA buffer. The total protein concentration was determined using an Enhanced BCA Protein assay kit (Beyotime). Briefly, protein electrophoresis was performed by using 10% SDS-PAGE, and then transferred to PVDF membrane (Invitrogen). Then, the PVDF membrane was probed with the primary antibody at 4°C overnight, and incubated with the secondary antibody (1:10,000) for 2 h at room temperature. β-actin was used as a loading control. The membrane was visualized using the chemiluminescence system. Supplementary Table S1 listed antibodies used in this study.

### RNA interference (RNAi)

Small interfering RNA (siRNA) fragment was synthesized by Generay Biotech (Shanghai, China). Cells were seeded in 6-well plates until they reached 80% confluence for transfection of siRNA with Lipofectamine® 2000 reagent according to the manufacturer's protocols. siRNA targeted to SLC25A39 and negative siRNA were supplied by General Biology (Chuzhou, China). siRNA sequences were listed in Supplementary Table S2.

### Cell apoptosis assay

Cell apoptosis was detected by Annexin V-FITC Kit (Beyotime, Shanghai, China) according to the manufacturer's protocol. In total, cells were harvested and resuspended with binding buffer, and stained with Annexin V-FITC and PI protected from light. Flow cytometry (Beckman, Fullerton, CA) was applied to analyze cell apoptosis.

### CCK8 assay and colony formation assay

Cells were plated into 96-well plates and routinely cultured. Cells were incubated with 10 μL CCK8 reagent at 37°C for 2 hours in the dark. The plate was read at 450 nm by a microplate reader (Bio-Rad, Hercules, CA, USA). Cells were seeded into a 12-well plate. Colonies were fixed with ice-cold methanol and stained with crystal violet after 2 weeks.

### Wound healing assay

RKO and DLD-1 cell migration was assessed by wound healing assay. Briefly, cells were seeded into a 6-well cell plate and incubated until reaching 80% confluence. Then, a scratch wound was applied in each well using a sterile pipette tip, and this time was made at 0 h. Cells were washed with PBS for several times and then incubated in medium without serum. Images of the cells were taken at 0 h and 72 h to assess the migration ability, respectively.

### Transwell chamber migration assay

For transwell migration assays, transwell chambers were inserted into the wells of 24-well cell plates, and RKO and DLD-1cells resuspended with the FBS-free medium were added into the upper room. 700 μL cell medium with 10% fetal bovine serum was added into the lower room. 48 h later, cells in the upper chamber were erased with a cotton swab, and the cells migrated to the lower surface of the chamber were stained with 1% crystal violet. Migration ability was evaluated by counting the cell numbers in five random microscopic fields per well.

### Identification of differentially expressed genes (DEGs)

The 408 tumor samples were divided into low SLC25A39 expression and high SLC25A39 expression groups based on the median expression of SLC25A39. The “limma” package of R (version 3.5.1) was explored to screen DEGs between the low SLC25A39 expression and high SLC25A39 expression groups. The screening criteria: 丨Log_(2)_ FC >1丨 and adjusted *P*<0.01. The “Gdc volcano Plot” package of R was used to plot the volcano map to visualize the DEGs.

### Gene Ontology and Kyoto Encyclopedia of Genes and Genomes (KEGG) analyses

“clusterPrifiler”, “org.Hs.eg.db”, “enrich plot”, and “ggplot2” packages of R (version 3.5.1) were applied to enrich the functional terms of DEGs between low SLC25A39 expression group and high SLC25A39 expression group. In addition, adjusted *P*<0.01 was applied to screen the functional candidates.

### Gene set enrichment analyses (GSEA)

Curated sets v7.4 collections were obtained from the Molecular Signatures Database as the target sets with which GSEA was running by GSEA 4.2.1 software. The total bulk RNA-seq data was loaded into the GSEA, and the gene set with *P*<0.001 and False Discovery Rate, *q*<0.001 were identified as statistically significant differences.

### Detection of Reactive Oxygen Species (ROS)

Mitochondrial ROS (mitoROS) was detected by using a mitoSOX fluorescence probe (Invitrogen, USA), following the protocols described previously. ImageJ software (National Institutes of Health, NIH) was applied to process the images and quantify the fluorescence value.

### In vivo subcutaneous xenograft models

The right flank of four-week-old Balb/c nude mice (five per group) was subcutaneously injected with RKO cells (5×10^6^ cells per animal). One week later, siR-vivo™ siSLC25A39 (50 mg/Kg) was injected into the tumor region in the siSLC25A39 group, and siR-vivo™ control siRNA was injected in the si ctrl group twice a week. The tumor volume was measured every three days for four weeks. At the conclusion of the four-week treatment period, mice were euthanized using an overdose of pentobarbital sodium, followed by cervical dislocation, in accordance with approved protocols by the Ethics Committee of Shanxi Medical University (Approval No. 2022GL060). All procedures adhered to the guidelines outlined in the Council of Agriculture Guidebook for the Care and Use of Laboratory Animals.

### Tumor immune microenvironment and immunotherapy response prediction

The ESTIMATE algorithm of R (version 3.5.1) was utilized to calculate the ESTIMATE score, immune score, stromal score, and tumor purity. The list of 11 immune checkpoints was referenced from a published study. GSE35640 was a melanoma immunotherapy dataset, which contained 65 metastatic melanoma patients treated with MAGE3. GSE176307 was a urothelial cancer immunotherapy dataset, which contained 89 urothelial cancer patients treated with PD1/PDL1. IMvigor210 was a bladder cancer cohort treated with PDL1, which contained 348 bladder cancer patients. These datasets were acquired from the GEO database (https://www.ncbi.nlm.nih.gov/gds/) and aimed to investigate whether SLC25A39 expression could accurately predict the response to immunotherapy.

### Single-cell data acquisition and preprocessing

Single-cell RNA-sequencing profiles of three CRC samples were acquired from GSE178318. R package Seurat was applied to analyze the scRNA-seq data, and the data quality criteria include, 500 < nFeature_RNA < 6000, and percent.mt < 5. Principal Component Analysis (PCA) and ScoreJackStraw were applied to narrow appropriate dimensions. Finally, t-distributed stochastic neighbor embedding (t-SNE) was applied to visualize the single-cell data.

### Prediction of therapeutic sensitivity in patients with different expressions of SLC25A39

The ability of SLC25A39 expression to predict the responses to 198 drugs for chemotherapies, targeted therapies, or small molecular drugs was evaluated in this study. 50% inhibiting concentration (IC50) of the 198 drugs was calculated by the pRRophetic package of R (version 3.5.1), and the IC50 values were normally transformed in this study. The detailed information on the drugs was obtained from Genomics of Drug Sensitivity in Cancer (GDSC, http://www.cancerrxgene.org/).

### Statistical analysis

All statistical analyses were performed using SPSS17.0 software (SPSS, Inc., USA). Data are presented as mean ± SD from three independent experiments. Student's t-test was applied to analyze the differences between the two groups. The correlations between variables were analyzed by Spearman's correlation. *P* > 0.05 was considered a statistically significant difference.

## Results

### SLC25A39 is up-regulated in CRC

**Figure 1:**
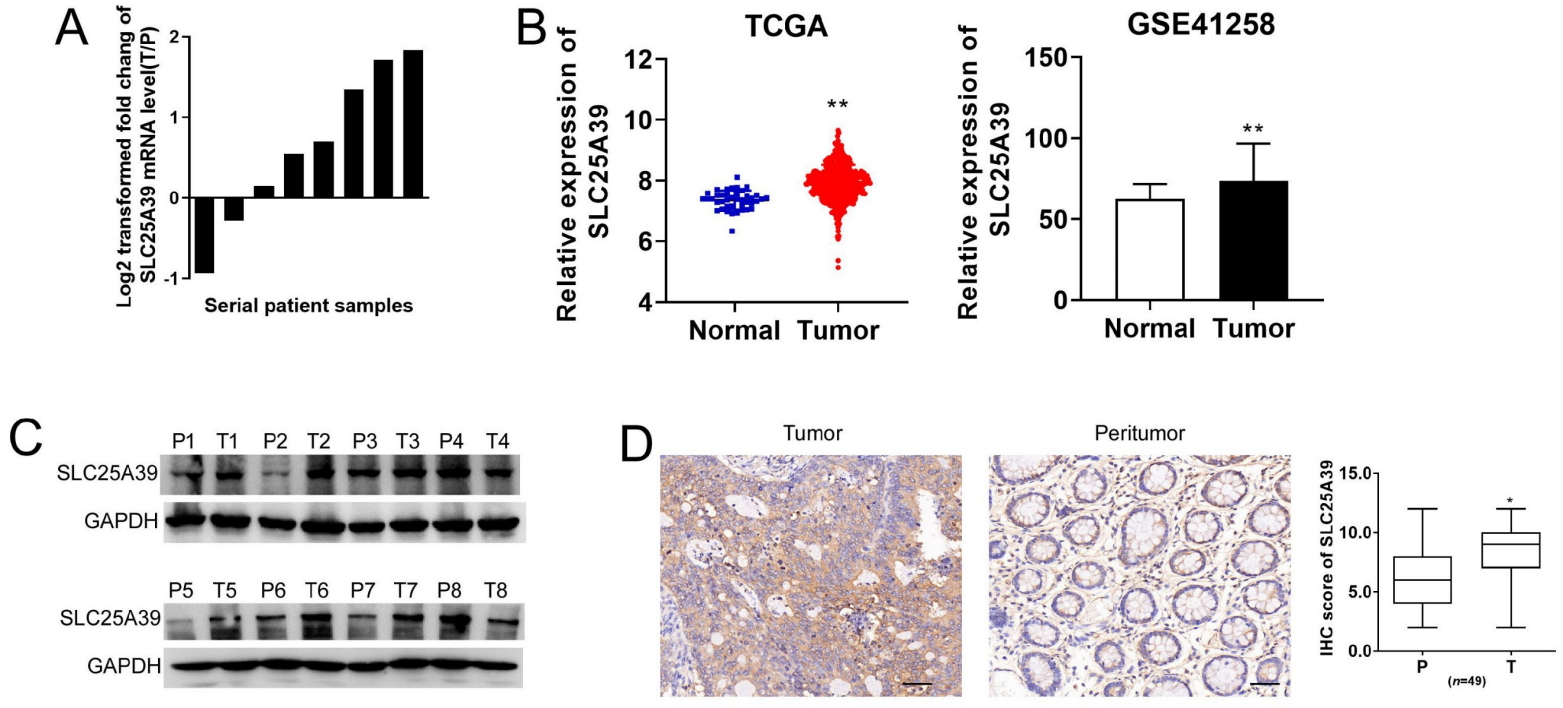
SLC25A39 is upregulated in colorectal carcinoma. (**A**) The relative mRNA expression ratio (Log2 transformed) of tumor/peritumor for SLC25A39 by qRT-PCR in 8 pairs of CRC tissues. (**B**) The relative mRNA expression levels of tumor and peritumor of SLC25A39 were analyzed in TCGA-COAD and GSE41258 public data. **(C)** Western blot analyses of SLC25A39 expressions in 8 paired tumors and adjacent normal tissues from CRC patients. (**D**) Representative IHC staining images (left panel) and IHC scores (right panel) of SLC25A39 in 49 paired tumor and peritumor tissues from CRC patients. scale bar, 50 µm. *P < 0.05; **P < 0.01; T, tumor; N, normal; IHC, immunohistochemical.

To elucidate the functional role of SLC25A39 in CRC, we initially assessed its expression level in 8 paired CRC tissues and adjacent non-tumorous tissues using qRT-PCR and western blotting assays. Our findings revealed a significantly higher mRNA expression of SLC25A39 in CRC tissues (Fig. 1A). To further delineate the expression pattern of SLC25A39 in CRC, we analyzed its expression in TCGA-COAD and GSE41258 datasets. Consistently, both datasets corroborated our qRT-PCR results, demonstrating elevated SLC25A39 expression in CRC tissues (Fig. 1B). The clinical characteristics of the low- and high-expression of SLC25A39 subgroups were then compared, and the difference of Gender (P=0.029) among the two subgroups reached statistical significance in TCGA-COAD datasets (Supplementary Table S3). Furthermore, the protein level of SLC25A39 were investigated in CRC tissues compared to adjacent normal tissues. Our western blot analysis indicated a notable increase in SLC25A39 protein expression in CRC tissues (Fig. 1C). To further validate the heightened expression of SLC25A39 in CRC, we conducted immunohistochemistry (IHC) analysis in 49 paired CRC and adjacent peritumor tissues. Our results demonstrated a significant elevation in SLC25A39 expression levels in CRC tissues (Fig. 1D and Fig. S1). In addition, the expression of SLC2539 was not associated with the clinical characteristic in our cohort (n=49, Supplementary Table S4). Collectively, these findings underscored the upregulation of SLC25A39 in CRC tissues.

### SLC25A39 promotes CRC cell growth in vitro

**Figure 2:**
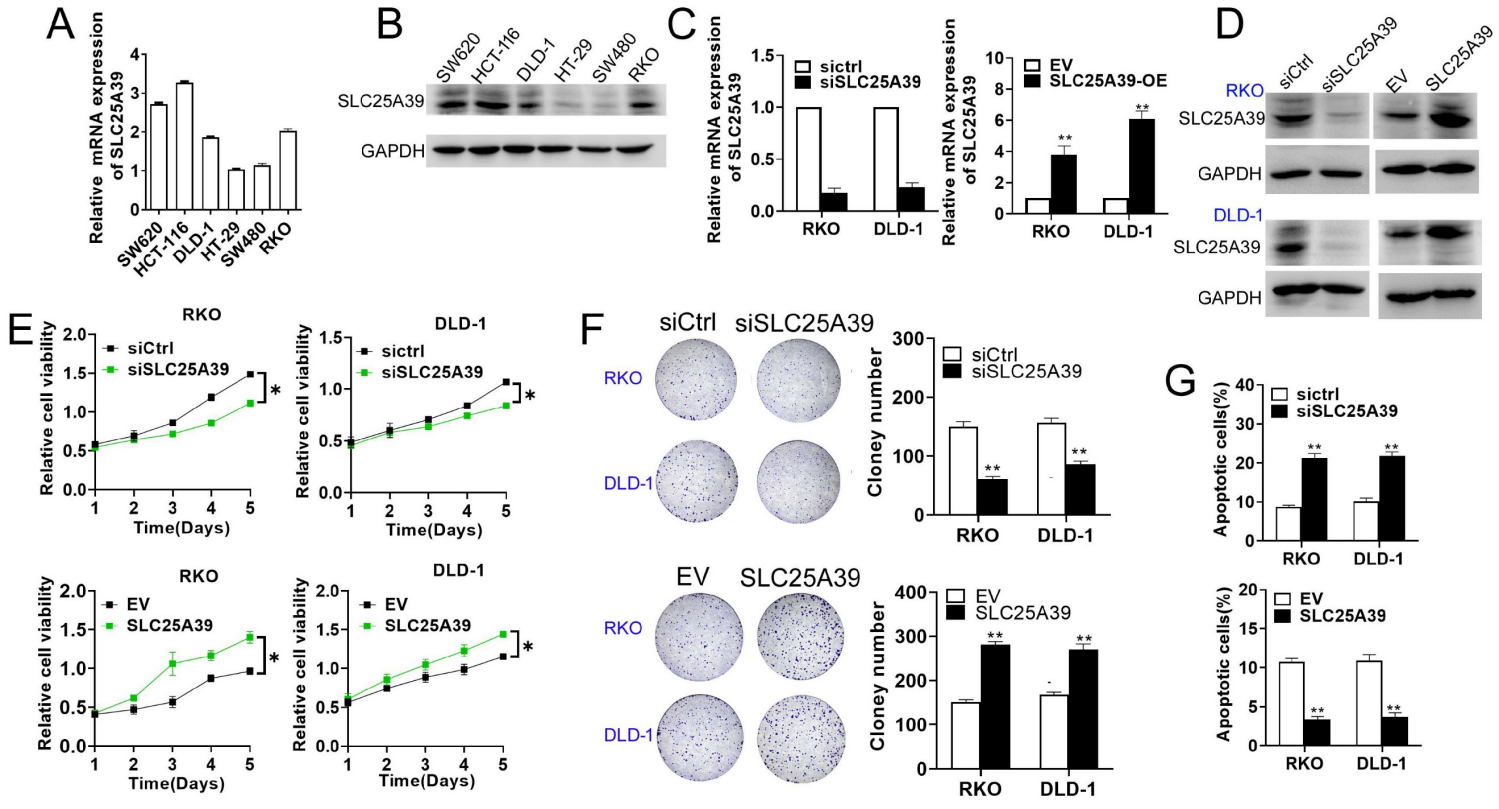
Up-regulation of SLC25A39 promotes CRC growth in vitro. (**A**) and (**B**) The mRNA and protein expression levels of SLC25A39 in different CRC cell lines (SW620, HCT116, DLD-1, HT-29, SW480, and RKO). (**C**) qRT-PCR analysis to measure SLC25A39 mRNA expression in RKO and DLD-1 cells, treated as indicated. **(D)** Western blot analysis to measure SLC25A39 protein levels in RKO and DLD-1 cells, treated as indicated. (**E**) CCK8 assays of RKO and DLD-1 cells, were treated as indicated. (**F**) Colony assays of RKO and DLD-1 cells, treated as indicated. (**G**) Flow cytometry analysis of cell apoptosis by Annexin-V and PI staining in RKO and DLD-1 cells, treated as indicated. *P < 0.05; **P < 0.01; SLC25A39, solute carrier family 25, member 39; siCtrl, negative control siRNA; siSLC25A39, siRNA against SLC25A39; EV, empty vector.

To explore the potential roles of SLC25A39 in CRC progression, we initially determined the its effect on cell growth in RKO and DLD-1 cells, representative of CRC cell lines with median SLC25A39 expression levels among six different types of CRC cell lines (Fig. 2A and B). In addition, the efficiency of SLC25A39 knockdown and overexpression were confirmed using qRT-PCR and western blot analyses (Fig. 2C and D). Our cell viability assay revealed that knockdown of SLC25A39 inhibited the growth of CRC cells compared to controls, whereas upregulation of SLC25A39 expression resulted in the opposite effect (Fig. 2E). Consistent with these findings, the number of colonies formed by CRC cells significantly decreased upon SLC25A39 knockdown, while overexpression of SLC25A39 led to an increase in colony formation (Fig. 2F). Furthermore, flow cytometry analysis demonstrated that knockdown of SLC25A39 increased the rate of cell apoptosis compared to controls, whereas overexpression of SLC25A39 exhibited the opposite effect (Fig. 2G). Collectively, these results indicate that SLC25A39 promotes the survival of CRC cells in vitro.

### SLC25A39 promotes CRC cell migration

**Figure 3:**
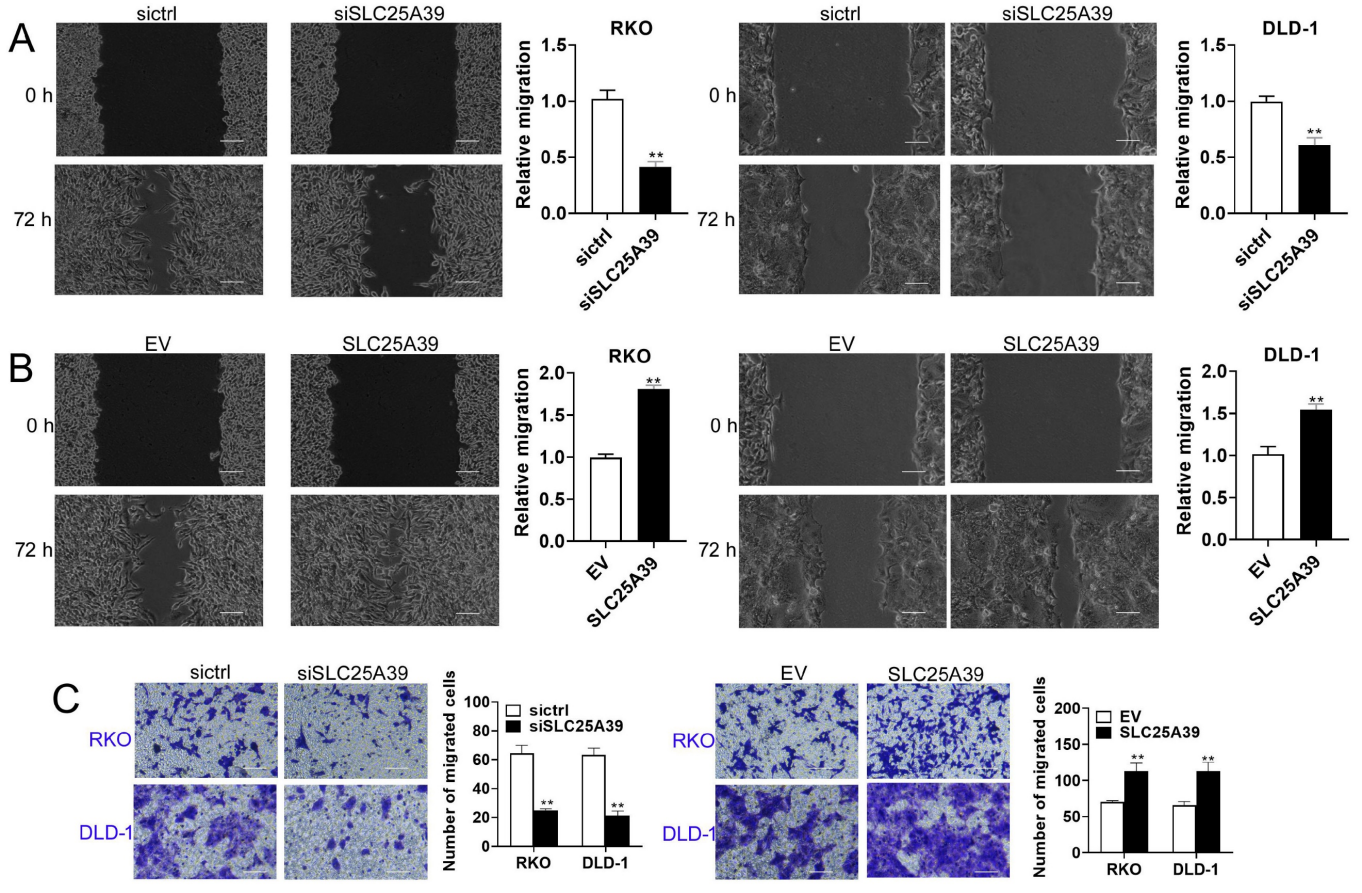
Up-regulation of SLC25A39 enhances CRC migration in vitro. (**A**) and **(B)** Scratch wound healing assays of RKO and DLD-1 cells, treated as indicated. **(C)** Transwell migration assays of RKO and DLD-1 cells, were treated as indicated. scale bar, 50 µm. **P < 0.01. SLC25A39, solute carrier family 25, member 39; siCtrl, control siRNA; siSLC25A39, siRNA against SLC25A39; EV, empty vector.

Next, we assessed the impact of SLC25A39 on CRC cell migration using scratch wound healing and transwell chamber assays. The scratch wound healing assay revealed that knockdown of SLC25A39 significantly impaired the migratory capacity of CRC cells, whereas overexpression of SLC25A39 resulted in enhanced migration (Fig. 3A and 3B). Similarly, the transwell chamber assay corroborated these findings, demonstrating that overexpression of SLC25A39 markedly increased CRC cell migration, while knockdown of SLC25A39 had the opposite effect (Fig. 3C). Collectively, these results indicate that SLC25A39 plays a pivotal role in promoting cell migration in CRC.

### Functional enrichment analysis of the DEGs between high SLC25A39 expression and low SLC25A39 expression groups

**Figure 4:**
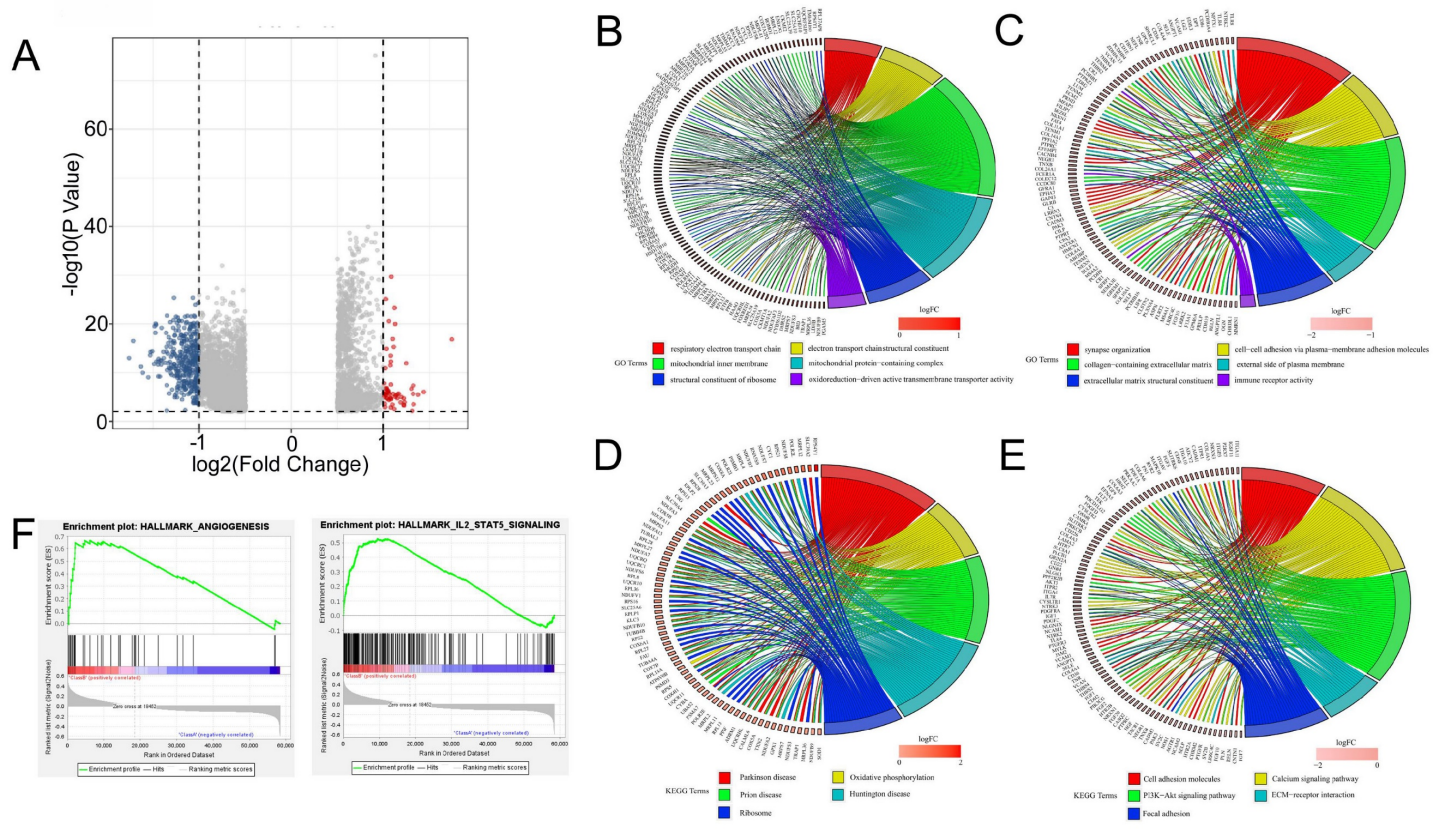
Enrichment functional analysis in high SLC25A39 expression group and low SLC25A39 expression group. (**A**) Volcano plot of differential analysis between high SLC25A39 expression group and low SLC25A39 expression group. (**B**) and (**C**) Circle maps. Bands with different colors in the right half circle symbolized 6 significant GO categories, including biological process (BP), cellular component (CC), and molecular function (MF). The 6 categories were enriched by genes listed in the left half circle. (**D**) and (**E**) Circle maps. Bands with different colors in the right half circle symbolized the top 10 significant KEGG pathways. The top10 pathways were enriched by genes listed in the left half circle. (**F**) GSEA recognized different gene sets in the high SLC25A39 expression group.

Furthermore, we conducted functional enrichment analyses of the 4903 DEGs between the high SLC25A39 expression group and low SLC25A39 expression group, comprising 1551 up-regulated genes and 3352 down-regulated genes (Fig. 4A and Supplementary Table 1 and Supplementary Table 2). GO enrichment analysis revealed that the up-regulated DEGs annotated to biological processes (BP) categories were mainly involved in cellular respiration, electron transport chain, and respiratory electron transport chain. The up-regulated DEGs annotated to cellular component (CC) categories were primarily linked to mitochondrial inner membrane, mitochondrial matrix, and mitochondrial protein-containing complex. The up-regulated DEGs annotated to molecular function (MF) categories highlighted the involvement of upregulated DEGs in structural constituent of ribosome, electron transfer activity, and primary active trans-membrane transporter activity (Fig. 4B and Fig.S2A). Conversely, downregulated DEGs were enriched in BP categories related to synapse organization, extracellular matrix organization, and extracellular structure organization. CC categories highlighted associations with collagen-containing extracellular matrix, neuronal cell body, and external side of the plasma membrane, while MF categories were linked to extracellular matrix structural constituent, immune receptor activity, and cytokine binding (Fig. 4C and Fig. S2B).

KEGG analysis revealed distinct pathways associated with upregulated and downregulated DEGs. The top pathways based on upregulated DEGs included chemical carcinogenesis, reactive oxygen species, ribosome, and oxidative phosphorylation, among others (Fig. 4D and Fig. S2C). Conversely, pathways enriched with downregulated DEGs encompassed PI3K-Akt signaling, calcium signaling, cytokine-cytokine receptor interaction, cell adhesion molecules, focal adhesion, phagosome, ECM-receptor interaction, cholinergic synapse, hematopoietic cell lineage, and malaria (Fig. 4E and Fig. S2D).

Furthermore, Gene Set Enrichment Analysis (GSEA) indicated significant associations between SLC25A39 expression and angiogenesis, IL2-STAT5 signaling, and IL6-JAK-STAT3 signaling in the high SLC25A39 expression group (Fig. 4F). Additional GSEA results for both high and low SLC25A39 expression groups are presented in Fig. S2E.

### Up-regulated SLC25A39 attenuates ROS accumulation in CRC cells

**Figure 5:**
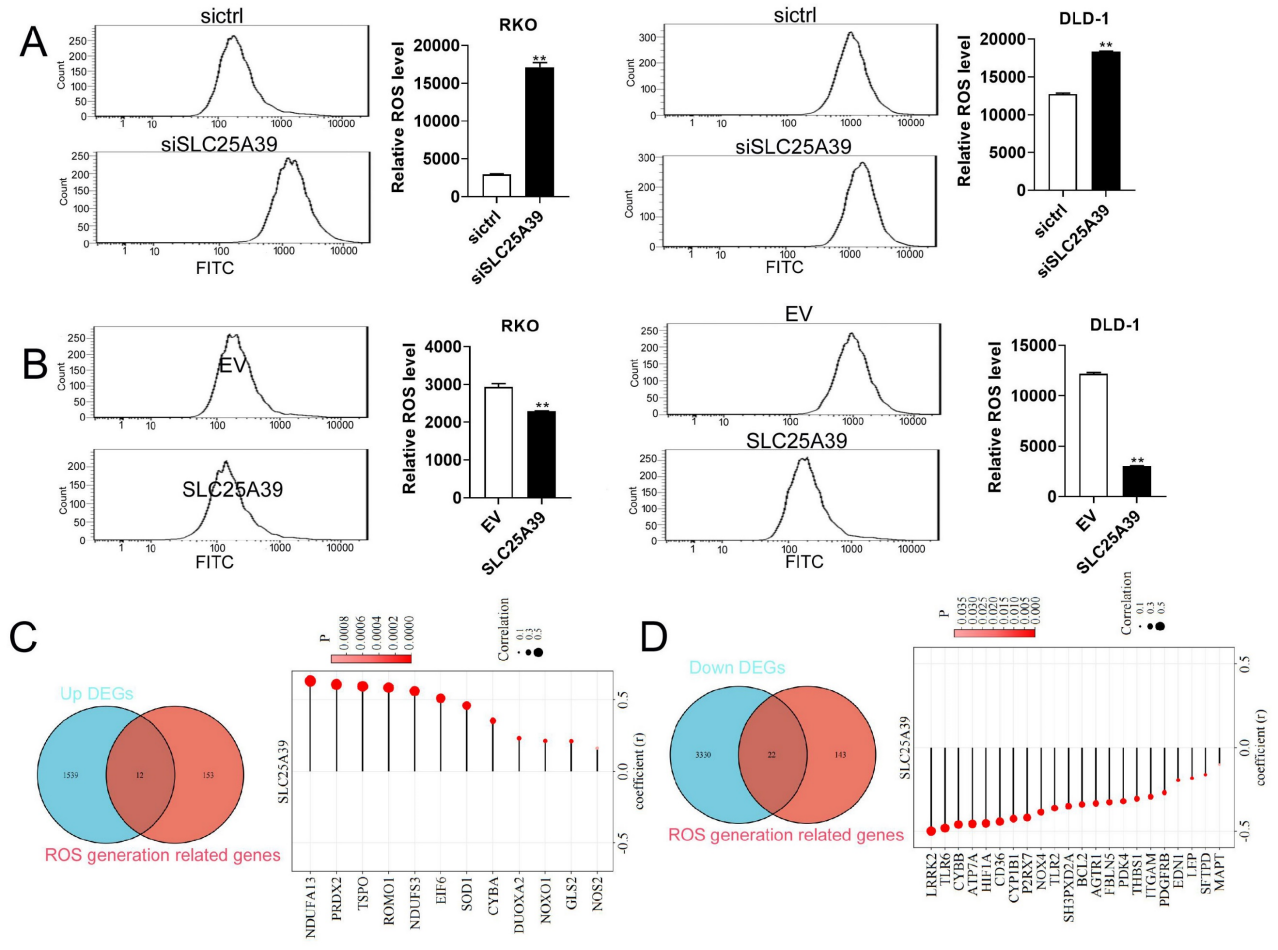
Up-regulation of SLC25A39 attenuates ROS accumulation. (**A**) and (**B**) ROS measured using the MitoSOX assay in RKO and DLD-1 cells, treated as indicated. (**C**) Venn diagram showed that the overlap of 1551 up-regulated DEGs and 165 ROS generation-related genes led to 12 hub genes being identified (left); Correlation analysis for SLC25A39 expression and the expressions of the 12 ROS generation-related hub genes(right); (**D**) Venn diagram showed that the overlap of 3352 down-regulated DEGs and 165 ROS generation-related genes led to 22 hub genes being identified (left); Correlation analysis for SLC25A39 expression and the expressions of the 22 ROS generation related hub genes(right); **P < 0.01.SLC25A39, solute carrier family 25, member 39; siCtrl, control siRNA; siSLC25A39, siRNA against SLC25A39; EV, empty vector; ROS, reactive oxygen species.

ROS play important roles in cell growth, migration, and apoptosis 15, 16. Moreover, ROS signaling pathway was enriched by KEGG analysis in the present study. Therefore, we investigated whether SLC25A39 contributed to CRC cell growth and migration via regulating ROS production. Our results revealed that knockdown of SLC25A39 significantly increased ROS level in CRC cells, while overexpression of SLC25A39 markedly decreased ROS level in CRC cells (Fig. 5A and B). To further explore the molecular mechanism underlying SLC25A39-mediated ROS regulation, we compiled a list of 165 ROS-associated genes from published literatures 17, 18 (Supplementary Table 3). Subsequently, combined analysis of these genes with the 1551 upregulated DEGs identified in our study led to the identification of 12 candidate ROS-associated upregulated DEGs in COAD, including NDUFA13, PRDX2, TSPO, ROMO1, NDUFS3, EIF6, SOD1, CYBA, DUOXA2, NOXO1, GLS2, and NOS2. Importantly, the expression of SLC25A39 was significantly and positively correlated with the expression of all 12 ROS-associated upregulated DEGs in COAD (Fig. 5C). Similarly, combined analysis of the 165 ROS-associated genes with the 3352 downregulated DEGs revealed 22 candidate ROS-associated downregulated DEGs in COAD, including LRPK2, TLR6, CYBB, ATP7A, HIF1A, CD36, CYP1B1, P2RX7, NOX4, TLR2, SH3PXD2A, BCL2, AGTR1, FBLN5, PDK4, THBS1, ITGAM, PDGFRB, EDN1, LEP, SFTPD, and MAPT. Notably, the expression of SLC25A39 was significantly and negatively correlated with the expression of all 22 ROS-associated downregulated DEGs in COAD (Fig. 5D). Taken together, our findings indicate that upregulated SLC25A39 promotes CRC cell growth and migration by reducing ROS accumulation. However, while we identified 22 ROS-associated DEGs in COAD, the precise molecular mechanism by which SLC25A39 regulates ROS remains to be fully elucidated and warrants further investigation in future studies.

### SLC25A39 promotes CRC growth in vivo

**Figure 6:**
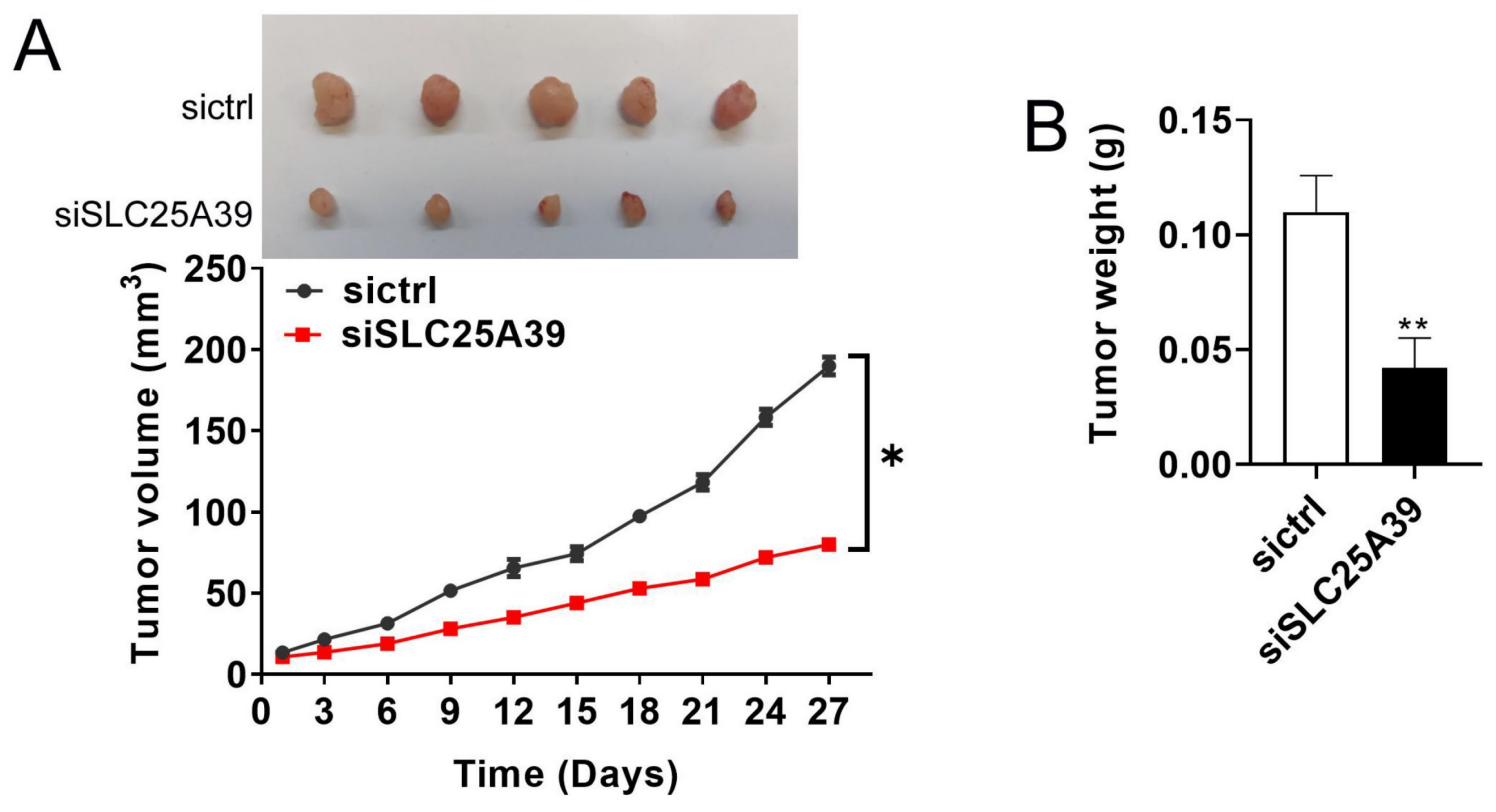
SLC25A39 promotes CRC xenograft growth in vivo. (**A**) Subcutaneous tumor growth curves and dissected tumors from sacrificed mice were shown, and treated as indicated in nude mice. (**B**) The tumor weight was determined after tumor nodules were harvested. *P<0.05; **P<0.01. siCtrl, control siRNA; siSLC25A39, siRNA against SLC25A39.

The effect of SLC25A39 on CRC growth was further studied in vivo by establishing a CRC xenograft nude mice model. As depicted in Fig. 6A, CRC xenografts with SLC25A39 knockdown exhibited a slower growth rate compared with the controls. Moreover, the tumor weight was significantly lighter in the CRC xenografts with SLC25A39 knockdown group compared with the controls (Fig. 6B). Taken together, these results indicated that SLC25A39 accelerated CRC growth in vivo.

### SLC25A39 expression was associated with tumor immune microenvironment and immunotherapy response in CRC

**Figure 7:**
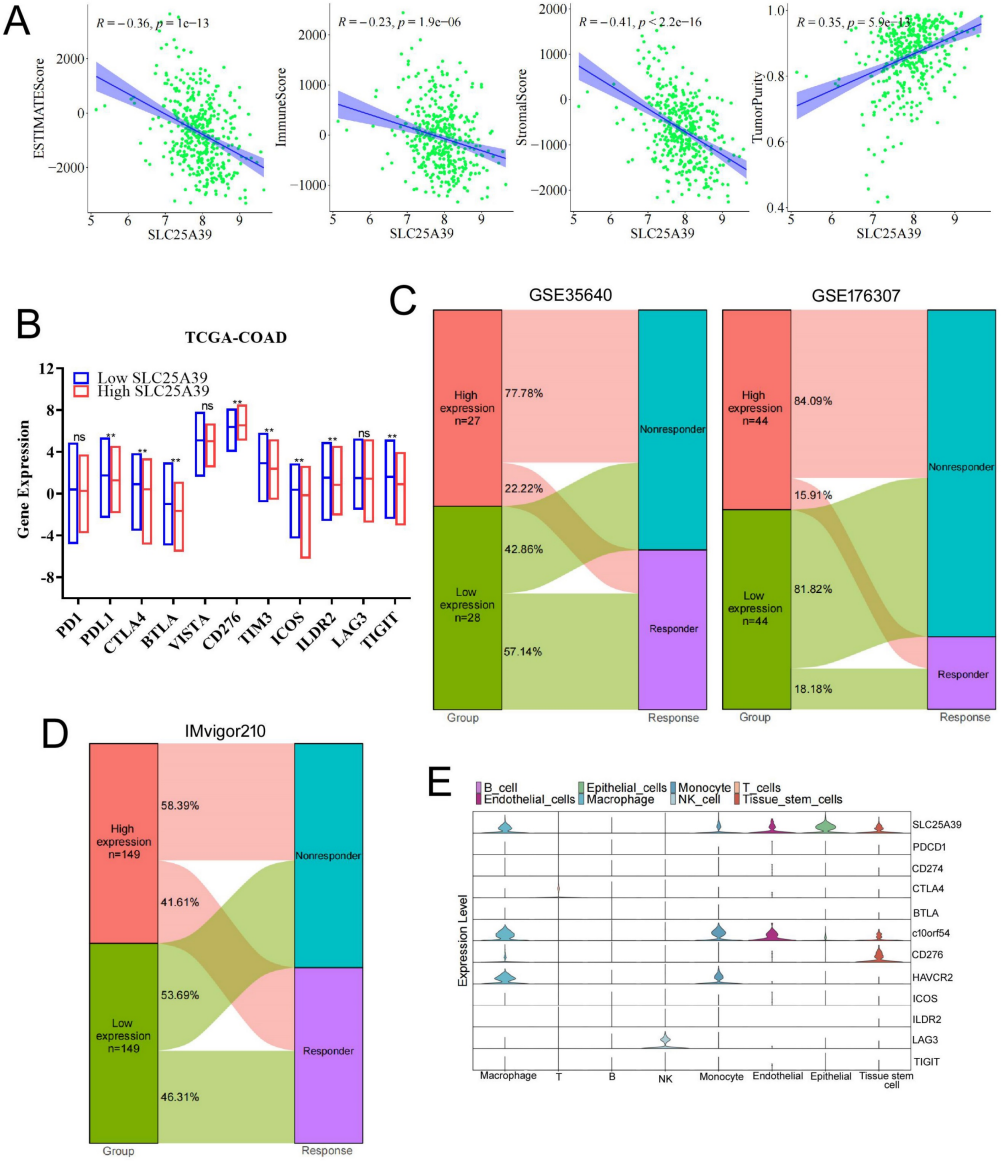
The associations of SLC25A39 expression of tumor immune microenvironment and immunotherapy response. (**A**) The correlation between estimate score, immune score, stromal score, and tumor purity and SLC25A39 expression in TCGA-COAD dataset, respectively. (**B**) Expression variation of 11 immune checkpoint points. **(C)** The proportion of patients with response to immunotherapy in low SLC25A39 expression group and high SLC25A39 expression groups in the GSE35640 immunotherapy cohort (65 metastatic melanoma patients received MAGE3 inhibitor treatment), and in the GSE176307 immunotherapy cohort (89 urothelial cancer patients received PD1/PDL1 inhibitor treatment). (**D**) The proportion of patients with response to immunotherapy in low SLC25A39 expression group and high SLC25A39 expression groups in the Mvigor210 immunotherapy cohort (348 bladder cancer patients received PDL1 inhibitor treatment). (**E**) Violin plot showing the expressions of 11 immune checkpoint points in GSE178318. **P<0.01.

The tumor immune microenvironment (TIME) was closely linked with the therapeutic response and prognosis. Therefore, it is rational to explore the association between the expression of SLC25A39 and the immune cell infiltration in CRC. The ESTIMATE results showed that CRC patients with high expression of SLC25A39 had lower immune score, lower stromal score, estimate score, and significantly higher tumor purity, than those in patients with low expression of SLC25A39 (*P*<0.001, Fig.7A). Nowadays, immune checkpoint points were studied and well applied in immunotherapy. In the present study, our results presented that 8 classical immune checkpoint points were considerably modulated in high SLC25A39 expression group. The expression of CD276 was significantly up-regulated in the high SLC35A39 group. In contrast, the expressions of PDL1, CTLA4, BTLA, TIM3, ICOS, ILDR2, and TIGIT were significantly decreased in high SLC35A39 group (Fig.7B).

Next, GSE35640 cohort (treatment with MAGE-A3 immunotherapeutic in metastatic melanoma patients) was applied to validate whether the expression of SLC25A39 could accurately predict the immunotherapy response. We found that the immunotherapy response rate in high SLC25A39 expression group (22.22%) was remarkably lower than in low SLC25A39 expression group (57.14%) in GSE35640 dataset. Consistent with the above results, the immunotherapy response rate in high SLC25A39 expression group (15.91%) was also lower than in low SLC25A39 expression group (18.18%) in GSE176307 dataset (A urothelial cancer cohort treated with PD1/PDL1, Fig.7C). Consistently, the similar results were observed in the IMvigor210 cohort (A bladder cancer cohort treated with PDL1, Fig.7D).

Next, single-cell RNA-Seq data was applied to analysis the expression of immune checkpoint points in different cell types. As shown in Fig. 7E and Fig.S3, SLC25A39 was mainly expressed in epithelial cells (colon adenocarcinoma cells), macrophage and tissue-stem cells, which suggested that SLC25A39 might also be involved in regulation of the cellular behavior of macrophage and tissue-stem cells. In addition, C10orf54 and HAVCR2 were mainly expressed in macrophage and monocyte, which indicated that combined knockdown of SLC25A39 in CRC cells and chimeric antigen receptor myeloid-cell immunotherapy targeted to C10orf54 or HAVCR2 might be an effective strategy in CRC immunotherapy. C10orf54 was reported to be involved in several processes, including negative regulation of cytokine production; positive regulation of macromolecule metabolic process; and regulation of T cell activation. HAVCR2 is a Th1-specific cell surface protein that regulates macrophage activation, and inhibits Th1-mediated auto- and alloimmune responses, and promotes immunological tolerance. Moreover, LAG3 was mainly expressed in NK cells, which indicated that combined knockdown of SLC25A39 in CRC cells and chimeric antigen receptor NK-cells immunotherapy targeted to LAG3 might also be an effective strategy in CRC immunotherapy. The sequence data, exon/intron organization, and chromosomal localization all indicate a close relationship of LAG3 to CD4. These results demonstrated that combination of the SLC25A39 expression and specific immune checkpoint points could be a potential effective strategy for CRC immunotherapy.

### The expression of SLC25A39 predicts therapeutic benefits in CRC

**Figure 8:**
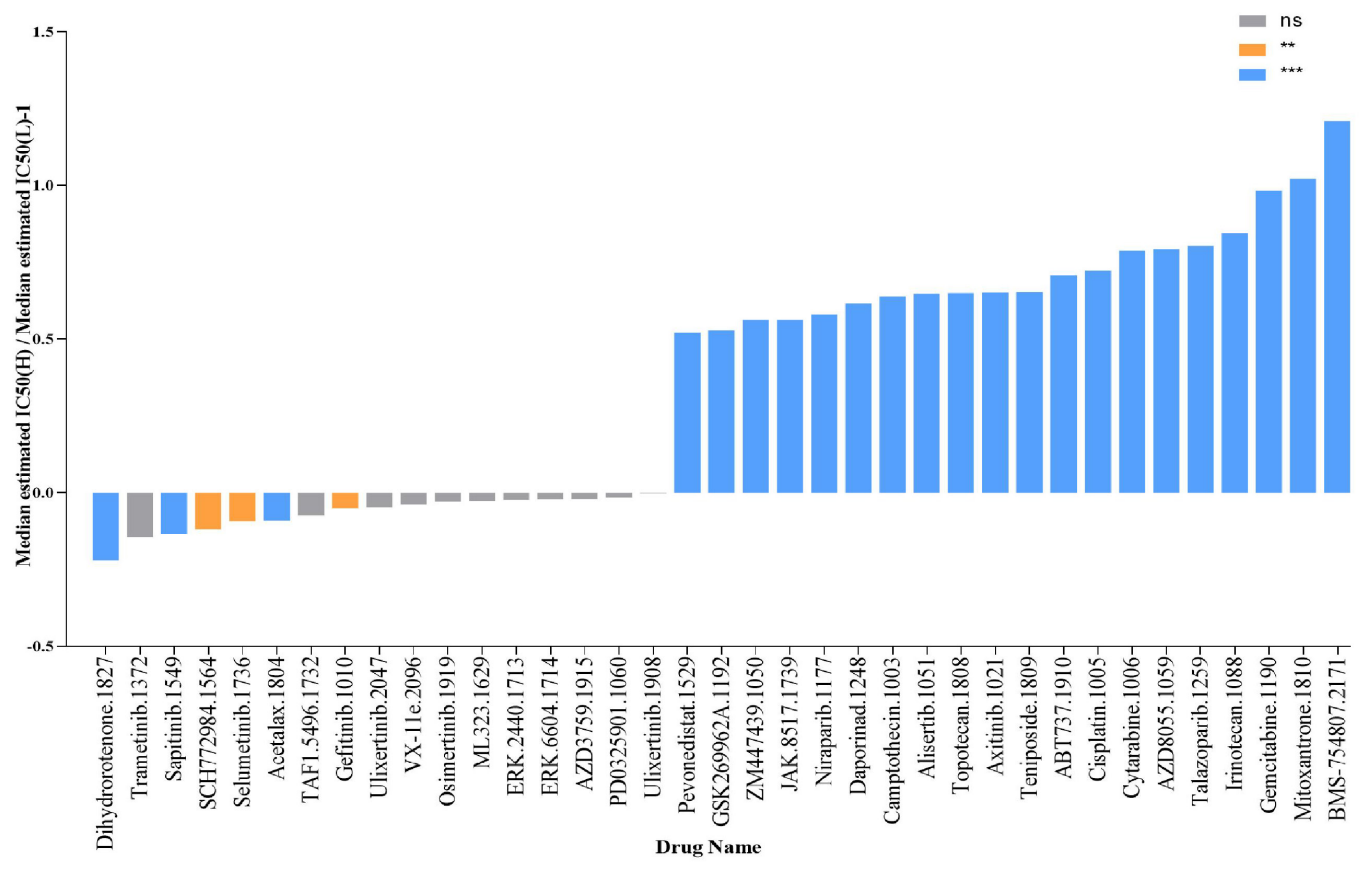
SLC25A39 expression predicts drug therapeutic benefits in COAD. The Proportion of normalized IC50 value of the 37 drugs between the low SLC25A39 expression group and high SLC25A39 expression group. *P* values were showed as: ns not significant; **P<0.01; ***P<0.001.

To evaluate the predictive power of SLC25A39 expression for drug response in CRC, including chemotherapy, targeted therapy, small molecule drugs, and immunotherapy, we calculated the IC50 values of 198 drugs in CRC. Our findings revealed that patients with high SLC25A39 expression were more sensitive to Dihydrorotenone, Trametinib, Sapitinib, SCH772984, and Selumetinib, whereas patients with low SLC25A39 expression exhibited sensitivity to BMS-75480, Mitoxantrone, Gemcitabine, Irinotecan, and Talazoparib. These results provide potential pharmacoeconomic guidelines for clinical treatment decisions (Fig. 8 and Fig. S4).

## Discussion

Mitochondria play a crucial role in maintaining optimal levels of metabolites necessary for both protective and biosynthetic functions. Among these, glutathione (GSH), a small-molecule thiol abundant in eukaryotes, is particularly vital for oxidative metabolism. Given mitochondria's central role in oxidative reactions, maintaining adequate GSH levels is essential for their protective and biosynthetic functions. Notably, GSH synthesis occurs exclusively in the cytosol. SLC25A39, responsible for importing mitochondrial GSH from the cytoplasm, emerges as a key player in safeguarding against oxidative stress and preserving mitochondrial function. Our research contributes to elucidating the functional significance of SLC25A39-mediated mitochondrial GSH in regulating the mitochondrial redox state.

Nowadays, the expression and prognostic significance of SLC25A39 in malignancies remain poorly understood, although extensive research has focused on its role in the nervous system. Within the central nervous system, fourteen novel members of the mitochondrial solute carrier family 25 (SLC25) are widely expressed, including SLC25A39 19. In our study, we found that SLC25A39 expression is up-regulated in colorectal cancer (CRC), with higher levels observed in male patients compared to female patients. Notably, elevated SLC25A39 expression correlates with poor survival in cases of HPV+ head and neck squamous carcinoma 20. Furthermore, mutations in SLC25A39 have been identified in sarcomas. Beddok's study, utilizing RNA-sequencing of five sarcoma tumors, revealed SLC25A39 mutations in all samples 21. Various factors have been reported to regulate SLC25A39 expression across different models. For instance, Atsushi Kawase et al. investigated the effects of cholestasis and lipopolysaccharide (LPS)-induced inflammation in mice, noting a decrease in Slc25a39 mRNA in the liver under cholestatic conditions, along with decreased Slc25a39/40 mRNA and protein levels in the kidneys. Consequently, there was a significant decrease in mitochondrial glutathione (mGSH) levels in the kidneys 22. Liu et al. demonstrated that SLC25A39 is rapidly degraded by the mitochondrial protease AFG3L2 under physiological conditions. Depletion of glutathione could dissociate AFG3L2 from SLC25A39, leading to a compensatory increase in mitochondrial GSH uptake 23. Additionally, Shi et al. revealed that human SLC25A39 is a short-lived protein under dual regulation at the protein level. Co-immunoprecipitation mass spectrometry and CRISPR knockout (KO) in mammalian cells identified mitochondrial m-AAA protease AFG3L2 as responsible for degrading SLC25A39 through the matrix loop 1. SLC25A39 senses mitochondrial iron-sulfur clusters using four matrix cysteine residues and inhibits its degradation. This dual regulation of transporters, by protein quality control and metabolic sensing, allows for modulation of mitochondrial glutathione levels in response to iron homeostasis, providing avenues for exploring the regulation of metabolic compartmentalization 24.

SLC25A39 serves as a pivotal regulator of glutathione (GSH) transport into mitochondria. Depletion of SLC25A39 diminishes mitochondrial GSH import and abundance, while cellular GSH levels remain unaffected. Cells deficient in both SLC25A39 and its paralogue SLC25A40 demonstrate compromised activity and stability of proteins containing iron-sulfur clusters. Furthermore, mitochondrial GSH import proves indispensable for in vitro cell proliferation and red blood cell development in mice. Notably, the availability of GSH negatively modulates SLC25A39 protein levels, thereby linking redox homeostasis to mitochondrial GSH import in mammalian cells. These findings underscore SLC25A39 as a crucial and regulated component of the mitochondrial GSH-import machinery 9. In a parallel vein, the knockdown of two candidate genes, SLC25A39 and TBC1D8, which lack substantial evidence of neuronal functions, results in decreased neurite outgrowth 25. Similarly, in our current investigation, we observed that the knockdown of SLC25A39 significantly impairs cell survival and migration, strongly implicating the reduction in mitochondrial GSH import mediated by SLC25A39 knockdown in inhibiting cell survival and migration in colorectal cancer (CRC).

Mitochondrial glutathione (GSH) plays indispensable roles in maintaining normal cellular functions, including protection against oxidative stress, as well as cell proliferation and division. It is distributed across various cellular compartments, with a notable presence in mitochondria (approximately 10-15%), the nucleus, and the endoplasmic reticulum 26. Augmenting the levels of the antioxidant GSH and the expression of SLC25A39 through the exogenous addition of glycine has been shown to mitigate lipid peroxidation induced by reactive oxygen species (ROS) and reduce the levels of malondialdehyde (MDA) by enhancing the functions of mitochondria, peroxisomes, and lipid droplets (LDs) 27. Consistent with these findings, our current investigation revealed that the knockdown of SLC25A39 resulted in heightened ROS accumulation, suggesting that the reduction in mitochondrial GSH import mediated by SLC25A39 knockdown impairs the cell's ability to effectively eliminate or neutralize ROS generated from mitochondria, thereby leading to increased ROS levels in colorectal cancer (CRC). Interestingly, a study reported that SLC25A39 mutant flies exhibited ROS accumulation alongside mitochondrial dysfunction, synaptic defects, and neurodegeneration 28. This underscores the critical role of SLC25A39-mediated mitochondrial GSH import in maintaining cellular redox balance and overall mitochondrial function.

Furthermore, alterations in glutathione homeostasis have been linked to both oxidative stress and mitochondrial damage 29. Nilsson R et al. demonstrated that the knockdown of SLC25A39 in murine erythroleukemia cells impaired iron incorporation into protoporphyrin IX, while a yeast mutant of the SLC25A39 gene exhibited an iron homeostasis defect, which could be restored by the vertebrate SLC25A39 30. Additionally, Gialluisi et al. identified sixteen novel candidate genes associated with late-onset Parkinson's disease, several of which are implicated in oxidative stress and mitochondrial metabolism, including SLC25A39 31. In our investigation, we present a novel finding: SLC25A39 plays a crucial role in cancer cell growth and migration. Despite the constant exposure of mitochondria to oxidant species, an effective antioxidant defense system, in which mitochondrial GSH (mGSH) plays a pivotal role, mitigates or repairs oxidative damage incurred during normal aerobic metabolism. Maintaining ROS homeostasis is essential for cell survival 32. Our data indicate that the down-regulation of SLC25A39 results in ROS accumulation, while up-regulation of SLC25A39 decreases ROS production. However, although our findings in the present study have considerable clinical implications, there are still some limitations. Firstly, the sample size of tumor tissues is small, more sample should be enrolled to validate the results presented in this study. Secondly, this study heavily relied on informatics analysis while the validation component is poor. More experiments are needed to validate in further studies. Finally, mechanisms of up-regulated SLC25A39 decreased ROS accumulation was not well characterized and need to be further explored.

## Conclusion

In summary, our findings underscore the essential role of SLC25A39 in promoting ROS-mediated colorectal cancer (CRC) growth and migration. Given its involvement in regulating mitochondrial glutathione (GSH) levels, both SLC25A39 and mitochondrial GSH emerge as potential therapeutic targets for CRC. Manipulating SLC25A39 function or mitochondrial GSH levels in tumor cells could induce selective oxidative stress, ultimately leading to cell death. Furthermore, variations in SLC25A39 protein expression among cancer cells may hold prognostic significance for patients, warranting further investigation to ascertain its potential as a cancer biomarker. Additionally, the development of agents targeting SLC25A39 or antibodies that specifically block its channel activity holds promise for therapeutic intervention in cancer metastasis.

## Supplementary Material

Supplementary figures and tables.
