# Supplementary material for: Up-regulated SLC25A39 promotes cell growth and metastasis via regulating ROS production in colorectal cancer
Source: J Cancer. 2024 Sep 16;15(17):5841–54. doi: 10.7150/jca.98844 (PMC11414614; doi:10.7150/jca.98844)
Supplement: Supplementary file 1 — Supplementary figures and tables. [file jcav15p5841s1.pdf]

Up-regulated SLC25A39 promotes cell growth and metastasis via regulating ROS production in colorectal cancer

Wentao Zhang[1,#], Zhigao Ou[1,#], Ting Tang[1], Tian Yang[1], Yubo Li[1], Hao Wu[1], Li, Li[1], Ming Liu[1], Li, Niu[2,*], Jianjun Zhu[3,*]

[1]Department of Medical Cellular Biology and Genetics, School of Basic Medical Science, Shanxi Medical University, Taiyuan, Shanxi, China

[2]Department of Pathophysiology, School of Basic Medical Science, Shanxi Medical University, Taiyuan, Shanxi, China

[3]Department of Medical Cellular Biology and Genetics, School of Basic Medical Science, Shanxi Medical University, First Hospital of Shanxi Medical University, Taiyuan, Shanxi, China

#Wentao Zhang and Zhigao Ou contributed equally to the work.

Corresponding authors: Li Niu, E-mail:nltys2004@163.com; Department of Pathophysiology, School of Basic Medical Science, Shanxi Medical University, Taiyuan, Shanxi, China. Jianjun Zhu, E-mail: zhujianjun@sxmu.edu.cn; Department of Medical Cell Biology and Genetics, Shanxi Medical University,Taiyuan, Shanxi, China.

Table S1. Primary antibodies used for immunohistochemistry and western blot.

Antibody	Company (Cat. No.)	Working dilutions
SLC25A39	Novus (NBP1-59600)	WB: 1/1000 IHC: 1/200
GAPDH	Proteintech (60004-1-lg)	WB: 1/3000

Table S2. Sequence of primers

1. **Primers used in qPCR**

SLC25A39	Forward primer	TCGTGAAGATCGTGAGGCAC
	Reverse primer	GGCTCGACCACACAGGAAG
β-actin	Forward primer	ACTCTTCCAGCCTTCCTTCC
	Forward primer	TCTCCTTCTGCATCCTGTCG

2. **siRNA**

SLC25A39	sense	ACUGUGCCAGCUACCGCCAUCUACU
	antisense	AGUAGAUGGCGGUAGCUGGCACAGU

Table S3. Clinical characteristics between low- and high-expression of SLC25A39 groups in TCGA-COAD

Factors	n	SLC25A39 Lower expression(204)	Higher expression(204)	c2	P value
**Gender**					
Male	220	99	121	4.774	**0.029**
Female	188	105	83		
**Age(years)**					
＜60	116	63	53	1.205	0.272
≥60	292	141	151		
**T stage**					
T1+T2	81	41	40	0.015	0.901
T3+T4+TX	327	163	164		
**N stage**					
N0	238	111	127	2.582	0.108
N1+NX	170	93	77		
**Distant metastasis**					
M0	309	147	162	3.001	0.083
M1+MX	99	57	42		
**Tumor stage**					
Stage 1+2	230	108	122	1.953	0.162
Stage 3+4	178	96	82		

Table S4. Clinical characteristics between low- and high-expression of SLC25A39 groups in self-acquired cohorts

Factors	n	SLC25A39 Lower expression(24)	SLC25A39 Higher expression(25)	c2	P value
**Gender**					
Male	29	14	15	0.014	0.905
Female	20	10	10		
**Age(years)**					
＜60	18	8	10	0.234	0.628
≥60	31	16	15		
**T stage**					
T1+T2	17	11	6	2.576	0.109
T3+T4+TX	32	13	19		
**N stage**					
N0	19	7	12	1.829	0.176
N1+NX	30	17	13		
**Distant metastasis**					
M0	31	18	13	2.787	0.095
M1+MX	18	6	12		
**Tumor stage**					
Stage 1+2	25	13	12	0.186	0.666
Stage 3+4	24	11	13		

Supplementary Figure and Figure legends

Figure S1

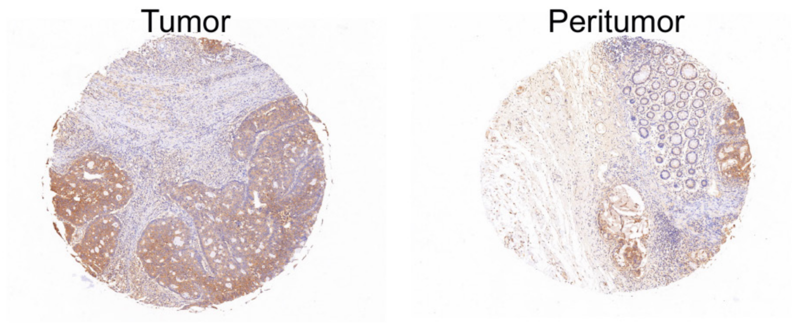

Figure S1. SLC25A39 is upregulated in colorectal carcinoma.

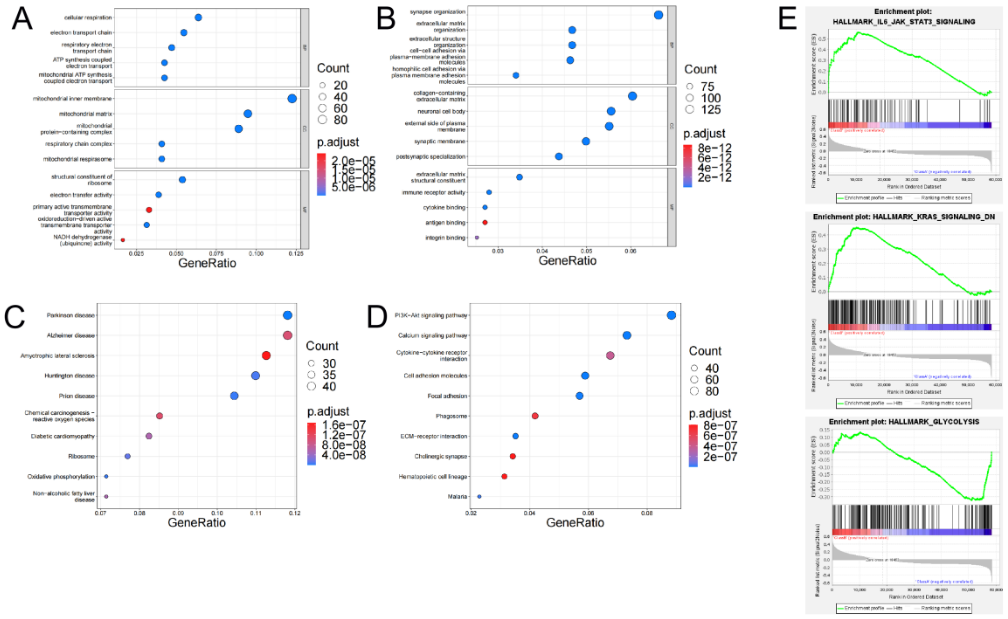

Fig. S2 Enrichment analysis in high SLC25A39 expression group and low SLC25A39 expression group. **(A)** GO analysis based on the up-regulated DEGs between the high SLC25A39 expression group and low SLC25A39 expression group. **(B)** GO analysis based on the down-regulated DEGs between the high SLC25A39 expression group and low SLC25A39 expression group. **(C)** KEGG analysis based on the up-regulated DEGs between the high SLC25A39 expression group and low SLC25A39 expression group. **(D)** KEGG analysis based on the down-regulated DEGs between the high SLC25A39 expression group and low SLC25A39 expression group. **(E)** GSEA recognized different gene sets in the high SLC25A39 group.

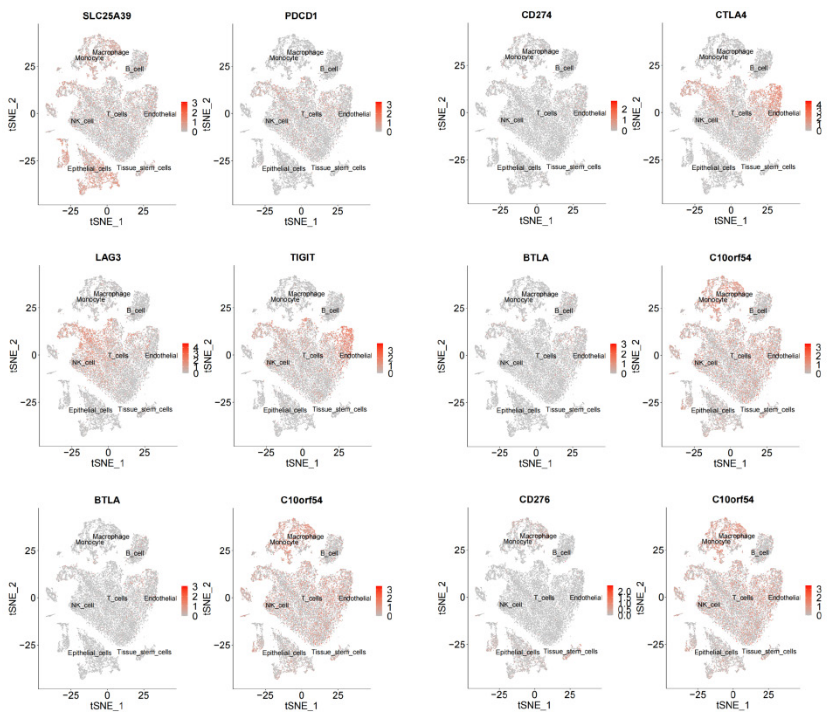

Fig. S3 The associations of SLC25A39 expression of tumor immune microenvironment and immunotherapy response.

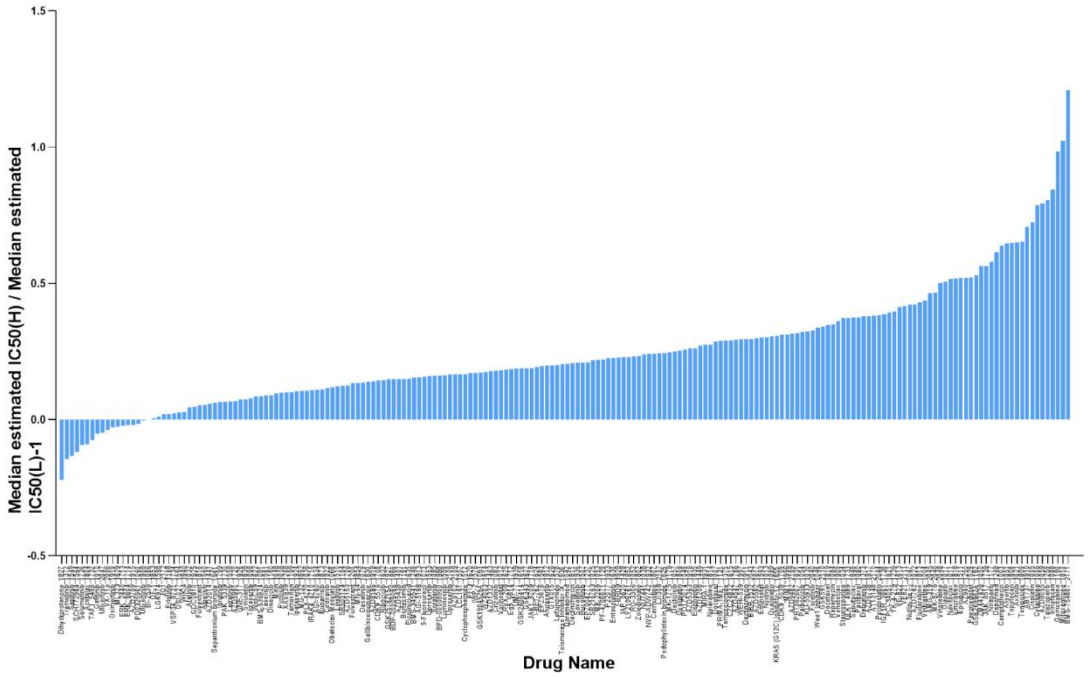

Fig. S4 SLC25A39 expression predicts drug therapeutic benefits in COAD. The Proportion of normalized IC50 value of the 198 drugs between the low SLC25A39 expression group and high SLC25A39 expression group.
